# Acceptance and Commitment Coaching for Music Performance Anxiety: Piloting a 6-Week Group Course With Undergraduate Dance and Musical Theatre Students

**DOI:** 10.3389/fpsyg.2022.830230

**Published:** 2022-03-18

**Authors:** Sarah E. Mahony, David G. Juncos, Debbie Winter

**Affiliations:** ^1^Performers College, Corringham, United Kingdom; ^2^Voice Study Centre, East Bergholt, United Kingdom; ^3^LifeStance Health, Philadelphia, PA, United States

**Keywords:** music performance anxiety (MPA), acceptance and commitment coaching, acceptance and commitment therapy, psychological flexibility, group coaching, performing arts students, vocal performance anxiety

## Abstract

Treatments for students with problematic levels of music performance anxiety (MPA) commonly rely on approaches in which students are referred to psychotherapists or other clinical professionals for individual care that falls outside of their music training experience. However, a more transdisciplinary approach in which MPA treatment is effectively integrated into students’ training in music/performing arts colleges by teachers who work in consultation with clinical psychologists may prove more beneficial, given the resistance students often experience toward psychotherapy. Training singing teachers, and perhaps music teachers at large, to use an evidence-based coaching strategy like Acceptance and Commitment Coaching (ACC) to directly manage students’ MPA is one such approach. Building on the work of a previous study in which ACC was administered by a singing teacher to a musical theatre student with problematic MPA, we piloted the effectiveness of a six-session, group ACC course for a sample of performing arts students (*N* = 6) with MPA related to vocal performances, using a mixed-methods design. The coach here was also a singing teacher without a clinical background, and her training in ACC by a clinical psychologist was of a similar duration (8 h) as the previous teacher’s (7 h). Similar to the musical theatre student, the students reported being significantly less fused with their MPA-related cognitions, more accepting of their MPA-related physiological symptoms, and more psychologically flexible while performing in general, and these improvements were maintained after 3 months. Furthermore, they appeared to lower their shame over having MPA and change how they thought in relation to one another. Of note, these improvements were similar to those shown by seven vocal students with MPA after they received Acceptance and Commitment Therapy from a clinical psychologist, but with larger reductions in shame and better acceptance of MPA, which suggests a non-clinical, group ACC intervention that includes supportive discussions to normalize MPA and challenges attempts to control it may be more helpful than individual psychotherapy. These results are promising and indicate a brief training in ACC (<10 h) may be sufficient for singing teachers to provide significant benefit for students with problematic MPA.

## Introduction

### Music Performance Anxiety in University Settings

Music performance anxiety (MPA) is a form of social anxiety that commonly affects professional and student musicians. MPA symptoms can be categorized into cognitive symptoms, physiological arousal symptoms, behavioral symptoms (which include both avoidant behaviors and anxious behaviors), and distress/impairment over having MPA - for examples of each symptom category, see Shaw et al. (2020). The more categories of MPA symptoms a musician experiences, the more problematic their MPA will be. Given their younger age, university music students are more likely to experience problematic levels of MPA, because they often have less experience performing at elite levels compared to professionals (Papageorgi et al., 2013; Biasutti and Concina, 2014). Students in competitive music and performing arts schools are also sensitive to their comparisons to other students, and to comparisons to their own self-standards, when self-evaluating the quality of their music performances (Denton and Chaplin, 2016). Thus, prevalence estimates for university students with problematic levels of MPA are purported to be slightly higher than those for professional musicians (20–35% for students vs. 15–25% for professionals; Fishbein et al., 1988; Wesner et al., 1990; Schroeder and Liebelt, 1999; Kaspersen and Gotestam, 2002).

Available treatments for music students with MPA vary but can be classified into three general categories: (1) *medication-based treatments*, such as beta-adrenoceptor blocking agents aka “beta-blockers” (Nube, 1991), and benzodiazepines (James and Savage, 1984), (2) *psychotherapy-based treatments*, such as Cognitive Behavioral Therapy (Kenny and Halls, 2018), Acceptance and Commitment Therapy (Juncos et al., 2017), and Psychodynamic Therapy (Kenny et al., 2014), and (3) *alternative treatments that either promote relaxation and improved physical health*, such as biofeedback (Wells et al., 2012), yoga (Khalsa et al., 2009), meditation (Lin et al., 2008), hypnosis (Brooker, 2018), and the Alexander Technique (Hoberg, 2008), *or are expressive arts therapies*, such as music therapy (Montello et al., 1990), guided imagery alone (Esplen and Hodnett, 1999), and guided imagery with progressive muscle relaxation (Sisterhen, 2005). Typically, these treatments are administered by psychologists and mental health clinicians with training in psychotherapy for anxiety disorders. However, music students may not choose to work with a psychotherapist due to stigma and lack of time/access to therapy, and rather, they may prefer to consult with their teachers about how best to cope with MPA (Williamon and Thompson, 2006; Shaw et al., 2020). A few of the aforementioned treatments indeed have been administered by music teachers and showed promising results, i.e., Alexander Technique (Hoberg, 2008), and guided imagery with progressive muscle relaxation (Sisterhen, 2005). These studies highlight the growing interest in enlisting music teachers’ participation in helping students manage MPA, as such an alternative treatment model may help to overcome the aforementioned hurdles preventing music students from seeking psychotherapy for MPA (Patston, 2014; Shaw et al., 2020).

### Music Teachers as Music Performance Anxiety Practitioners

Given the close bond between music teachers and students, it is unsurprising music students may prefer to consult with them, rather than with a psychotherapist, about how to manage their MPA. One-on-one lessons afford students the privacy and individualized attention they would receive in psychotherapy, but without the stigma. It is inevitable that psychological issues related to a student’s performances, and possibly issues of a more personal nature, will arise under such conditions, especially if the teacher-student relationship exhibits similar qualities known to correlate with good psychotherapy outcomes. Shaw et al. (2020) rightly point out such *relational* qualities known to correlate with effective psychotherapy outcomes also exist within the teacher-student dyad, i.e., a relationship marked by empathy, congruence, and unconditional positive regard, and a good working alliance marked by an agreement on the goals to be achieved and the methods to achieve them (Rogers, 1951; Bordin, 1979; Karver et al., 2006; Ardito and Rabellino, 2011). Thus, music teachers who notice these qualities in their relationships with students are likely to make a positive impact when individually coaching one with problematic MPA, and teachers who haven’t yet noticed these qualities will want to prioritize building up the rapport and working alliance with a student(s) before starting this work. Of course, when faced with students’ more personal issues unrelated to music performance, teachers must recognize them as falling more within the jurisdiction of a psychotherapist, and appropriate referrals must be made regardless of the strength of the relationship.

Classroom teachers also deal with a myriad of emotional and behavioral issues and are often encouraged to seek training in psychological methods to better equip them to handle such issues. One common example is Applied Behavioral Analysis, or “ABA,” which is an evidence-based framework for addressing the needs of students with Autism and other neuro-developmental disorders, such as AD/HD (Cook et al., 2014; Makrygianni et al., 2018). With training in ABA, classroom teachers may cut down on students’ disruptive behaviors, improve IQ scores, communication skills, adaptive behavior, and their social skills, with training ranging from 1.5 h to multi-day workshops - and teachers who participate are able to show a high proficiency in demonstrating specific ABA skills post-training (Sarokoff and Sturmey, 2004; Lerman et al., 2008; Makrygianni et al., 2018; Alberto et al., 2022). Other examples are the mental health training and certification program offered by the International Board of Credentialing and Continuing Education Standards (IBCCES) and the training in Mental Health First Aid offered in numerous countries, including the United States and United Kingdom (National Council for Mental Wellbeing, 2021). These programs offer training for special and general education teachers, and numerous other school staff, to help identify and support students struggling with common psychological problems, such as depression, anxiety, trauma, suicidal ideation, substance abuse, and others (International Board of Credentialing and Continuing Education Standards, 2021; National Council for Mental Wellbeing, 2021). Classroom teachers can learn evidence-based solutions for handling their students’ emotional and/or behavioral challenges, which helps to fill the training gap faced by many who regularly witness students’ psychological struggles yet feel unqualified to help (Jorm et al., 2010; Glasper, 2017; Diaz, 2018).

In light of the similarities between the music teacher-student/psychotherapist–client relationship, and the increasing trend of training classroom educators to better handle students’ mental health needs, it is important to start viewing music teachers as capable MPA practitioners, whether they work in the private studio, classroom setting, or both. While MPA is often understood as a problem occurring within the student, in that it reliably occurs in individuals who exhibit numerous risk factors for developing it, some of which include perfectionism, being female, age younger than 30, being classically trained (Mor et al., 1995; Sinden, 1999; Kenny, 2011; Kenny et al., 2012; Papageorgi et al., 2013), MPA is simultaneously occurring at a cultural level, in that it reliably occurs in contexts in which high standards for success are expected, e.g., conservatories and university music departments, competitive auditions for professional work. Thus, it would benefit music teachers – who already operate within these contexts – to receive training in managing MPA in order to offset the pressures facing students and to introduce a better balance into the training experiences offered by music and performing arts colleges. By outsourcing MPA treatment to psychotherapists, the problem is approached too idiographically and will continue to be perceived that way. Of course, psychological conditions must be treated at the individual level by psychotherapists with the requisite graduate-level education, adequate training, and a license to practice independently. However, problematic cases of MPA could be effectively managed institutionally, by teachers who work in music/performance arts colleges and are adequately trained by clinical psychologists to help students in such a transdisciplinary manner (Shaw et al., 2020).

### Group Coaching as an Intervention Strategy by Music Teachers for Students With Music Performance Anxiety

Music teachers who wish to address their students’ MPA but lack time for an individualized coaching approach (e.g., Shaw et al., 2020) may consider a group coaching intervention as an alternative that could impact a larger number of students. Group coaching is often discussed interchangeably with team coaching, but they are distinct. Team coaching seeks to address and change a team’s dynamics, enabling them to work toward an enhanced self-coaching capability, whereas group coaching includes people seeking individual outcomes whilst exploring them within a group setting (Clutterbuck, 2009). Thus, group coaching requires the coach to have an understanding of group dynamics and dialogue processes, along with the ability to create a rapport and connection with each individual (Brown and Grant, 2010). There are advantages of group interventions over individual ones, in that they are often more cost-effective and facilitate change via interactive group processes that don’t occur as readily, if at all, within individual settings, e.g., social comparison, social support, and providing supportive yet challenging feedback (Borek and Abraham, 2018). These and other helpful processes are even more likely to occur when groups are homogenous in their composition, mixed-gendered, and group sizes are small, i.e., five to seven members (Burlingame et al., 2003; Borek and Abraham, 2018).

Social comparison theory asserts that people self-evaluate through their comparisons to others, including *upward comparisons* to a group member of perceived higher status or skill and *downward comparisons* to someone of perceived lesser status or skill (Festinger, 1954). Such evaluations can be helpful when one’s comparisons (whether upward or downward) lead them to be influenced by their peers in a positive, motivative way, but they become unhelpful when the comparisons involve discrepancies in skill or group status that are either too large, or non-modifiable, and when used in this maladaptive way social comparisons correlate with shame, perfectionism, and psychopathology (Allan and Gilbert, 1995; Walton et al., 2020). Applied to MPA, this might involve a student making an upwards comparison to a peer with less outward anxiety and concluding her peer is a better performer than she is. Social support is the provision of psychological and/or material help to group members, and it is associated with improved physical and mental health (Thorsteinsson and James, 1999; Borek and Abraham, 2018). It may include providing members with helpful information, giving someone a positive appraisal of themselves, providing emotional support, or providing help in learning specific skills (Borek and Abraham, 2018). Receiving supportive, but challenging, feedback from a group facilitator or a peer(s) after making an important self-disclose is another important group process that may lead one to personal change.

While the relational qualities may be similar between and a therapist/client, teacher/student, and arguably between a coach/coachee, these professions are not the same, and thus, group coaching must be distinguished from classroom teaching and group psychotherapy, in order to reduce the potential for unethical behavior on part of the music teacher. Whereas teaching is imparting information through instruction, coaching involves unlocking one’s potential in order to maximize their performance (Whitmore, 2017). In classroom teaching, a student’s personal or performance goals would be less important than the teacher’s curriculum, however, identifying and working toward students’ goals would be prioritized in group coaching. Psychotherapy is distinct from coaching in that it often adopts a pathology-focused approach and aims to resolve one’s symptoms of psychological distress, whereas, coaching typically adopts a skills-focused approach and aims to enhance one’s existing skills to unlock their potential (Hart et al., 2001; Grant, 2003; Cavanagh et al., 2006). Classroom teachers must avoid the diagnosis and treatment of mental health problems and again must refer a student with known, or suspected, signs of a psychological disorder to appropriate psychotherapy providers.

### Acceptance and Commitment Coaching as a Non-clinical Music Performance Anxiety Treatment

**FIGURE 1:**
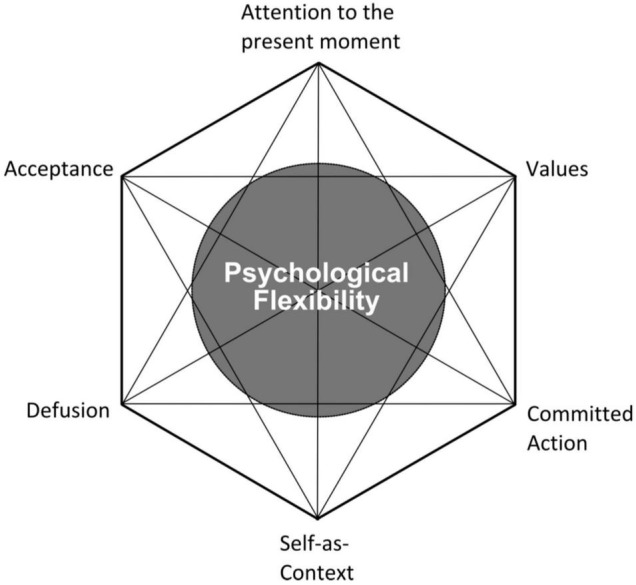
The ACT Hexaflex (Batink et al., 2016).

Recently, a non-clinical version of Acceptance and Commitment Therapy (or “ACT” – pronounced as the word “act”) was individually administered by a singing teacher to a male, musical theatre student to effectively treat his problematic MPA, and the results were promising (Shaw et al., 2020). When administered in non-clinical (non-psychotherapeutic) settings, ACT is called Acceptance and Commitment Coaching (ACC) or Acceptance and Commitment Training (also abbreviated as ACT and said as the word “act”). ACT (Hayes et al., 1999, 2011) is part of the “third-wave” of behavioral psychotherapies, which is also referred to as the “mindfulness and acceptance-based wave” of psychotherapies. As such a therapy, ACT does not aim to reduce symptoms of anxiety or emotional distress. Rather, it promotes mindfulness and acceptance of those symptoms while simultaneously increasing one’s commitment to behave in accordance with their personally held values. Such a dual skill set creates a more *psychologically flexible* behavioral repertoire, which is the overall aim of ACT and ACC (Hayes et al., 1999, 2011; Hill and Oliver, 2019). Paradoxically, a reduction of one’s anxiety or other symptoms of emotional distress usually does occur within ACT treatments, but it is not the direct goal (Bach and Moran, 2008). Applied specifically to the treatment of MPA, ACT fosters both mindfulness and acceptance of physiological MPA symptoms, defusing or disentangling oneself from cognitive MPA symptoms, learning to identity more as an observer of one’s MPA symptoms rather than taking them personally, identifying one’s performance-related values, and making the commitment to increase actions reflective of one’s values during performances, rather than over-engaging in avoidant or emotion-driven behavior (Juncos et al., 2014, 2017; Juncos and Markman, 2015). These six skills are collectively known as the “ACT Hexaflex” (Figure 1) and are thought to be ongoing behavioral processes that directly contribute to psychological flexibility (Hayes et al., 1999, 2011).

Shaw et al. (2020) compared their results in using ACC to those of previous ACT for MPA psychotherapy studies and found the musical theatre student who received the ACC intervention showed a nearly identical response as participants who received ACT psychotherapy (Juncos et al., 2014, 2017; Juncos and Markman, 2015; Clarke et al., 2020). That is, musicians in these studies appeared to become more accepting of their physiological MPA symptoms, less fused with their cognitive MPA symptoms, and less avoidant/more value-driven with their performance-related behavior, even while experiencing MPA. Further, the singing teacher who provided the ACC intervention had no formal education or training in psychotherapy, rather, she received approximately 7 h of individualized training by a clinical psychologist with expertise in using ACT to treat MPA. She also received post-graduate training in a popular coaching model, GROW (Goal, Reality, Options, and Will; Whitmore, 1992), that is widely used by executive coaches to improve work performance. The GROW model was used to provide further structure to the ACC intervention. The similarities in results between Shaw et al. (2020) study and those of previous ACT for MPA psychotherapy studies (Juncos et al., 2014, 2017; Juncos and Markman, 2015) suggest music teachers may be able to use ACC to effectively treat MPA in non-clinical settings, without needing to continually outsource this work to psychotherapists. If a non-clinical practitioner who is not a psychotherapist, e.g., a music teacher or performance coach, wishes to become competent in using ACT to treat MPA, they must first receive adequate training in it by a variety of means, i.e., attending online or in-person courses and/or workshops, individualized training from an ACT expert, extensive readings, peer consultation groups, or post-graduate study with ACT training included. The training should also include supervision from someone with expertise in the approach who can evaluate the practitioner’s core competencies and readiness to practice ACT. Unlike the Alexander Technique, ACT does not require a certification to be used by clinical or non-clinical practitioners, which may increase the number of practitioners aiming to use it.

### Group Acceptance and Commitment Coaching for Music Performance Anxiety

Thus far, no studies have examined a group ACC intervention administered by a music teacher to treat MPA, yet there are several reasons why it may prove beneficial. Two previous ACT for MPA studies involved a group therapy component, and both treatments led to significant improvements in psychological flexibility and reductions in MPA: Juncos et al. (2017) study included six group performances (in addition to 12 individual ACT sessions) in which the student vocalists performed in front of one another, whereas Clarke et al. (2020) study was entirely group-based and also had student vocalists perform in front of one another during one of their six sessions. The groups in both were of similar size (*N* = 7 in Juncos et al., 2017; *N* = 6 in Clarke et al., 2020). Moreover, group-based ACT psychotherapy treatments have shown similarly positive results in treating other anxiety disorders, including Social Anxiety Disorder (Kocovski et al., 2013), Generalized Anxiety Disorder (Avdagic et al., 2014), public speaking anxiety (England et al., 2012), and mixed anxiety disorders (Glaser et al., 2009). While individual ACC has already shown promising results when directly administered by a singing teacher to a student with problematic MPA (Shaw et al., 2020), a group ACC intervention may be more ecologically valid due to its cost-effectiveness.

From a group coaching perspective based on ACC, the coach would aim to mitigate some of the unhelpful processes arising for students with problematic MPA, like excessive fusion with cognitive MPA symptoms, a lack of willingness to perform with physiological arousal MPA symptoms present, and excessive behavioral avoidance/lack of values-guided action. These patterns of inflexibility were targeted in Juncos et al. (2017) and Clarke et al. (2020) treatments. Resolving these processes would include teaching students with MPA to identify how they are stuck in those patterns of behavior, while also improving the Hexaflex processes considered the healthier counterparts to those patterns, e.g., training in defusion helps lessen the harmful impact of excessive fusion with one’s MPA-related thoughts, increasing one’s willingness to perform with physiological MPA symptoms lessens the impact of excessive behavioral avoidance within one’s performances, and helping to identify one’s performance values and elicit a commitment from the student to behave more in accordance with them helps discipline one’s behavior so it is more values-based and less emotion-driven.

### Aims of the Current Study

Continuing with the research initiated by Shaw et al. (2020), this study aimed to preliminarily investigate the effectiveness of ACC as a non-clinical MPA intervention, also when administered by a singing teacher without training or education in psychotherapy. Here, the ACC intervention will be delivered via a group coaching course, rather than a single-subject methodology, to a small group of undergraduate dance and musical theatre students with homogenous levels of MPA related to their vocal performances skills. It should be noted this study did not aim to establish whether ACC has efficacy in treating MPA, as that would require a more well-controlled methodology. Rather, it aimed to determine whether a group ACC course would have ecological validity for use in music/performing arts colleges with students who experience problematic levels of MPA. ACT as a psychotherapy has already demonstrated efficacy in treating Social Anxiety Disorder (Kocovski et al., 2013; Craske et al., 2014; Herbert et al., 2018; Krafft et al., 2020), of which MPA would be considered a “performance-only subtype” according to the *Diagnostic and Statistical Manual of Mental Disorders - Fifth Edition* (American Psychiatric Association, 2013). Thus, it stands to reason that ACC might be generally helpful for students with MPA. A mixed-methods design with combined qualitative and quantitative data was selected to allow for a continuous and thorough assessment of students’ responses to the ACC course. The following five hypotheses were made for the study’s quantitative data:

1) It was predicted there would be significant improvements in some of the measurable ACC Hexaflex processes, i.e., *mindful acceptance* of MPA-related physiological symptoms, *defusion* from MPA-related thoughts, and improvements in overall *psychological flexibility*, at post-coaching compared to pre-coaching, and these improvements would be maintained at a 3-month follow-up.
2) The group ACC intervention would lead to significant improvements in students’ performance quality ratings at post-coaching when compared to their pre-coaching ratings, as adjudicated by three independent jurors.
3) Students’ shame over having MPA would be significantly reduced during their performances, and also more generally as indicated by significant improvements in their social comparisons at post-coaching, and at a 3-month follow-up.
4) Although ACC does not aim to reduce symptoms of MPA, it was hypothesized the students’ MPA levels would be significantly decreased at post-coaching, and at the follow-up, as previous ACT interventions have led to significant reductions in MPA (Juncos and Markman, 2015; Juncos et al., 2017; Clarke et al., 2020).
5) Lastly, the ACC intervention would yield clinically significant results similar to those observed after the ACT psychotherapy intervention was used in Juncos et al. (2017) with seven student vocalists undergoing training in classical singing. In other words, there would be visibly similar improvements on the total/subscale scores for questionnaires used here and in the 2017 study, i.e., BAFT, PHLMS, AAQ, KMPAI, and ESS.

## Materials and Methods

### Participants

Ethical approval for the study was obtained from the Institutional Review Board at University of Wales Trinity St. David (UWTSD). The participants were second-year Professional Musical Theatre and Dance students (*N* = 6) from Performers College, an independent college in the United Kingdom specializing in performing arts and also where the primary author (SM) works as a vocal tutor. Four of the students were specializing in Dance Theatre, whereas two were specializing in Musical Theatre. Students from both specializations train together, while some classes and shows are more focused on either specialization. Five of the students were enrolled in a Bachelor’s Degree track, and one was enrolled in a 3-year Diploma track. Four were female and two were male. Their ages ranged from 18-years-old to 20-years-old (*M* = 19.33, *SD* = 0.82).

Interested applicants responded to a screening assessment conducted online, and they were eligible to participate if they experienced distressing symptoms of MPA while singing, were in their second year of study, not currently receiving psychological or psychiatric treatment, not a current student of the primary author’s, and if their Kenny Music Performance Anxiety Inventory (KMPAI) score was above the clinical cutoff (105). Six students met inclusion criteria and provided their consent to participate in the study.

### Coach

SM served as the coach who delivered the ACC intervention. She was undertaking a Master’s degree in Voice Pedagogy with the Voice Study Centre, a provider of postgraduate study accredited by the UWTSD, and this research project made up her Master’s thesis. SM is trained in singing, acting, and musical theatre and has extensive vocal and instrumental performance experience. At Performers College, she teaches singing theory, technique, ensemble and solo singing, and she also maintains a private voice studio. Her work within higher education musical theatre training has led her to observe the debilitating effects of MPA on students firsthand, prompting her to research available MPA treatments and subsequently design an evidence-based, group coaching intervention for MPA that might readily be used by singing teachers of other student cohorts. Like the primary author in Shaw et al. (2020) study, she had no training or education in psychotherapy, and she received training in a popular coaching model, GROW (Whitmore, 1992), as part of her M. A. education. The third author (DW) helped her secure ethics board approval and provided supervision regarding some ethical matters inherent in coaching students with MPA, i.e., ensuring she was not functioning as a psychotherapist to the students, and that students were aware of available counseling resources through the British Association of Performing Arts Medicine (BAPAM). SM’s coaching course only aimed to treat the students’ MPA and did not target matters of a more personal nature.

### Procedure

#### Training

As in Shaw et al. (2020) study, SM was trained in ACC by the secondary author (DJ), a clinical and performance psychologist with 16 years’ experience in treating anxiety disorders and specific expertise in using ACT to treat MPA. DJ has conducted two single-subject designs with instrumentalists and a pilot study with vocal students (*N* = 7), in which ACT psychotherapy was investigated as a MPA treatment. He also provides training for singing and instrumentalist teachers, performances coaches, and other non-clinical professionals in using ACT/ACC to treat MPA and to enhance music performance. SM’s training in ACC lasted approximately 8 h – it included attending a 5-h ACT for MPA online course taught by DJ in May, 2020, an additional 3 h of individualized ACC training on Zoom with DJ, and regular email correspondence during the ACC intervention about how students were responding and specific guidance for those students who appeared to need extra help. Their scores on the ACC self-report measures at the mid-way point informed DJ which students needed further help and what type of exercises would be helpful.

#### Group Coaching Course

An ACC group course using Hill and Oliver’s (2019) book *Acceptance and Commitment Coaching: Distinctive Features* as a guide was created for the current study. This book features a six-session outline for coaches who wish to use ACC with clients (pp. 120–126), from which the course content was pulled. Additional content was taken from the secondary author’s book, *ACT for Musicians: A Guide for Using Acceptance and Commitment Training to Enhance Performance Skills, Overcome Performance Anxiety, and Improve Well-Being* (Juncos and de Paiva e Pona, 2022) and from various other ACT resources referenced below. The six sessions targeted the same ACT Hexaflex processes addressed in previous ACT for MPA psychotherapy studies (Juncos et al., 2017), i.e., mindfulness of MPA symptoms, increased willingness toward/acceptance of MPA symptoms, defusion from MPA-related thoughts, observing one’s MPA symptoms rather than defining oneself by them, identifying performance-related values, and committed action toward those values.

Regular exercises to increase mindfulness of MPA symptoms were used during sessions, e.g., “Mind watching” (Forsyth and Eifert, 2016), “Scanning a picture frame” (Juncos and de Paiva e Pona, 2022), “Hearing your thoughts” (Harris, 2009), and students were encouraged to practice at home in between sessions. Students strengthened willingness to have MPA symptoms and ultimately to become more accepting of them by discussing the futility of attempts to control MPA symptoms, and through group and individual exercises, e.g., “The difference between what I can and cannot control” (Forsyth and Eifert, 2016), and “Acceptance of performance-related thoughts and feelings” (Juncos and de Paiva e Pona, 2022). Defusion from MPA-related thoughts and unhelpful comparative thinking was taught through discussion, videos, and exercises, e.g., “Treating your mind like a separate entity and describing what it’s doing,” and “Thanking your mind for the thoughts it creates,” (Juncos and de Paiva e Pona, 2022). Self-as-context and learning to take MPA less personally were improved through discussion. Students’ performance-related values were identified through regular discussion, videos, and exercises, e.g., “Values checklist” (Harris, 2009), “Questions to to help identify your performance-related values (Juncos and de Paiva e Pona, 2022). Lastly, a commitment to performing more in accordance with students’ performance values was strengthened by regular discussion and group exercises, e.g., “Passengers on a bus metaphor” (Hayes et al., 2011) and creating S.M.A.R.T. goals. Overall psychological flexibility was strengthened by using the “ACT Matrix” tool (Polk and Schoendorff, 2014) within the group setting, e.g., by having students come up with examples of *away moves* in relation to their attempts to control/avoid MPA symptoms, and *toward moves* in relation to their engaging in committed actions more often during performances. Students were given regular homework assignments to further strengthen ACC skills in between sessions. For a detailed outline of the six sessions, readers are directed here: https://www.researchgate.net/publication/358271152_Acceptance_and_Commitment_Coaching_for_MPA_Course_Design_Sarah_Mahony.pdf.

In addition to ACC skills, students were taught to improve self-compassion for experiencing shame in relation to their MPA, and when overly engaged with unhelpful, comparative thinking, through group discussion, video, and group and individual exercises, e.g., “Working with difficult emotions,” “Working with shame” (Neff and Germer, 2018), and “Acceptance of performance-related thoughts and feelings” (Juncos and de Paiva e Pona, 2022). Lastly, students were made aware during the last session of several skills from a Psychological Skills Training protocol that may further help to manage MPA, i.e., visualization, positive self-talk, and breath control (Hardy et al., 1996; Spahn et al., 2016). However, these skills were not directly taught to them, given the different theoretical stance PST has (compared to ACC) toward controlling vs. accepting symptoms of physiological arousal.

The six sessions were conducted entirely online through Zoom, as the ACC group course took place in the United Kingdom during the COVID-19 pandemic (November to December, 2020). Their sixth session took place at the beginning of winter break, shortly after their term had ended. Sessions lasted 1 h each and their content was delivered via power-point slides. Students were sent an information handout after each session and encouraged to keep a journal of their experience with the course. All sessions were recorded and the video/audio data, along with students’ questionnaire data, was securely stored on SM’s laptop following General Data Protection Regulation standards.

#### Performance Schedule

Students video-recorded two vocal performances at pre-coaching (Session 1), in order to assess for baseline performance quality: one with accompaniment and one solo (a cappella). Similarly, they gave another accompanied and solo video-recorded performance at post-coaching (Session 6). Due to potential latency problems with performing music virtually on Zoom, and to the UK’s restrictions on social gatherings during the COVID-19 pandemic, their accompaniment was not live, rather, the students provided an instrumental back-up track for them to sing over. All students provided consent to be video-recorded. The primary author and other research participants served as each student’s audience members. Students were instructed to select four songs that demonstrated their highest current singing proficiency, to achieve consistency of performance, and to select different songs to minimize any potential practice effects. Immediately after each performance, each student was instructed to complete an ESS questionnaire and remain in the audience for their peers.

#### Music Performance Quality

Video-recordings for performances from each participant were randomized and sent to be marked by three expert judges. All three judges had significant experience with teaching and assessment within various educational settings, including professional musical theatre training colleges. Additionally, they were all professional musical theatre creative, with credits in performance, directing and musical direction. The judges did not know the purpose of the study, nor the order in which the recordings were presented. Additionally, they were asked to use their expertise to imagine the performances under real-life conditions and score accordingly. To avoid unfair marking based on circumstances out of the group’s control, judges were instructed not to take the following into account while scoring: appearance/outfit, lighting, camera angle, quality of video or sound, quality of internet connection, or location of performance.

The scoring criteria was adapted from the Associated Board of the Royal Schools of Music (ABRSM) singing exam marking scheme, a scoring criteria regularly used to adjudicate United Kingdom students’ vocal performances. Each domain was scored from 1 out of 10 points (where 1 = *poor*, and 10 = *excellent*) and included the following areas: *Pitch/Intonation, Tempo and Rhythm, Vocal Tone and Projection, Musicality (Dynamics, Vocal Expression), Character and Storytelling, and Confidence and Stage Presence*. All students provided consent to have their performances adjudicated.

### Self-Report Measures

#### Acceptance and Commitment Coaching-Based Process Measures

The Philadelphia Mindfulness Scale (PHLMS; Cardaciotto et al., 2008) was used to assess mindfulness. It is a 20-item measure comprised of two subscales: the Awareness subscale, which measures one’s continuous monitoring of internal and external experiences, and the Acceptance subscale, which measures one’s non-judgmental attitude toward one’s experiences. It uses a 5-point Likert scale, and higher subscale scores indicate higher levels of each construct. Internal consistency for the test is adequate for both the Awareness subscale (α = 0.75) and the Acceptance subscale (α = 0.82).

The Acceptance and Action Questionnaire-2 (AAQ-2; Bond et al., 2011) is a 7-item measure that was used to assess acceptance and psychological flexibility. It uses a 7-point Likert scale, and higher scores indicate higher levels of psychological flexibility and acceptance. Furthermore, the AAQ-2 shows good internal consistency (α = 0.84).

The Believability in Anxious Feelings and Thoughts (BAFT; Herzberg et al., 2012) was used to assess cognitive defusion, and it requires participants to indicate on a 7-point Likert scale how much they agree with each of 30 items. Higher scores on the BAFT reflect higher levels of fusion with one’s anxious thoughts and feelings. It shows excellent internal consistency (α = 0.90).

#### Symptom-Based Measures

The revised KMPAI (Kenny, 2009) is a 40-item measure used to assess psychological discomfort associated with MPA. It uses a 7-point Likert scale, and higher scores indicate greater levels of anxiety and MPA-related distress. The revised KMPAI shows excellent internal consistency (α = 0.94). Its author also suggests a score of 105 or higher indicates clinically significant MPA (Ackermann et al., 2014).

The Experiential Shame Scale (ESS; Turner, 2014) is a 11-item self-report measure that assesses the degree to which one identifies with physical, emotional, and social components of the emotion of shame, using a a 7-point Likert scale. Higher scores indicate higher levels of state shame. The last question was modified to reflect one’s willingness to discuss their music performance at the moment the ESS is taken. The ESS has adequate internal consistency (α = 0.72).

The Social Comparison Scale (SCS; Allan and Gilbert, 1995) assesses how one ranks themselves in relation to others, either favorably or unfavorably, in areas such as ability, inclusion, and desirability. Respondents rate how they feel about themselves on 11 dichotomous concepts using a 10-point, Likert scale. Higher scores indicate more favorable ratings compared to others. The SCS has excellent internal consistency (α = 0.91).

### Assessment Schedule and Qualitative Data Collection

Students completed the full battery of self-report measures (AAQ-2, BAFT, PHLMS, KMPAI, SCS) at baseline (Session 1), at the mid-way point (Session 3), at post-coaching (Session 6), and at a 3-month follow-up assessment. The KMPAI was also used as a screening measure, so each participant had two baseline KMPAI scores at the start of the ACC intervention. Students were also interviewed 1 week after the sixth session to assess their overall impressions of the coaching course and to elicit feedback about what might be done to improve it in the future. Interviews lasted 15 min each, followed a semi-structured format, and also took place/were recorded via Zoom. In total, the study’s qualitative data included verbatim transcripts of each of the six ACC sessions and interviews, along with written reflections at the sixth session and the follow-up.

## Results

### Changes in Psychological Flexibility

Within-group changes on mean scores for questionnaires measuring overall psychological flexibility (AAQ-II) and specific Hexaflex processes, i.e., defusion (BAFT), mindful acceptance and awareness (PHLMS *Acceptance* and PHLMS *Awareness*), were evaluated post-coaching and at the 3-month follow-up assessment, using paired *t*-tests. Significant improvements were observed within students’ pre-coaching (Session 1) mean scores and scores at post-coaching (Session 6) and at the 3-month follow-up assessment on all but one questionnaire (PHLMS *Awareness*), thereby supporting Hypothesis #1. See Table 1 for paired *t*-test results and *p* values for scores on these measures. Although no significant differences were observed within pre- and post-coaching (and at follow-up) scores on the PHLMS *Awareness* subscale, there was a trend toward improved mindful awareness at the follow-up assessment. Effect sizes were medium to large both at post-coaching and the 3-month follow-up point (see Table 2).

**TABLE 1:** Descriptive data and mean scores for ACC-based measures, as well as results of paired *t*-tests comparing pre-coaching means (Session 1) to means at post-coaching (Session 6) and at a 3-month follow-up.

Schedule	Self-Report Measures			
	*BAFT*	*PHLMS*	*PHLMS*	*AAQ-II*
		(Aware)	(Accept)	
	*M* = 50.1*^a^*	*M* = 36.65*^b^*	*M* = 30.19*^b^*	*M* = 17.34*^c^*
	*SD* = 16.88	*SD* = 4.93	*SD* = 5.84	*SD* = 4.37
S1 (Pre)	*M* = 82.83	*M* = 36.67	*M* = 20.67	*M* = 29.5
	*SD* = 16.1	*SD* = 6.53	*SD* = 6.09	*SD* = 8.14
S6 (Post)	*M* = 44	*M* = 38.17	*M* = 30.5	*M* = 22.67
	*SD* = 7.75	*SD* = 4.12	*SD* = 6.89	*SD* = 10.19
	*t*(5) = –6.97*	*t*(5) = 0.74	*t*(5) = 3.43*	*t*(5) = –3.88*
FU	*M* = 44.17	*M* = 41	*M* = 30.67	*M* = 23.5
	*SD* = 6.85	*SD* = 4.34	*SD* = 7.58	*SD* = 7.92
	*t*(5) = –6.1*	*t*(5) = 1.92	*t*(5) = 2.96*	*t*(5) = –2.88*

*Normative means and standard deviations for the ^a^BAFT, ^b^PHLMS, and ^c^AAQ-II, were taken from non-clinical samples of undergraduates used in each measure’s validation study.*

*Statistically significant result at p < 0.05 level compared to S1 mean.

*S, session; FU, follow-up.*

**TABLE 2:** Effect sizes for changes in scores on self-report measures from Session 1 to Session 6 and to 3-month follow-up, using Hedges’ *g*.

			Self-Report Measures				
	BAFT	PHLMS	AAQ-II	KMPAI*	SCS	ESS	ESS
		(Accept)				(Accomp.)	(A Cappella)
S6	*g* = 3.07	*g* = 1.52	*g* = 0.74	*g* = 1.87	*g* = 1.44	*g* = 2.35	*g* = 1.91
FU	*g* = 3.07	*g* = 1.46	*g* = 0.75	*g* = 2.48	*g* = 1.86	n/a	n/a

*KMPAI scores from Session 1 were used as a baseline comparison, rather than students’ scores at the screening assessment.

*S, session; FU, follow-up.*

### Changes in Music Performance Quality

The study’s second hypothesis, that the ACC intervention would lead to significant improvements in students’ performance quality, was not supported by the three judges’ ratings. A repeated measures ANOVA was conducted with performance time (pre vs. post-coaching) and raters (Judges 1, 2, or 3) as within-subject variables, and judges’ total ABRSM scores as the dependent variable. There was no significant main effect of time on the three judges’ total scores: *F*(1,5) = 0.65, *p* = 0.46, nor were there significant main effects of time on the judges’ scores for accompanied vs. a cappella performances: *F*(1,5) = 0.17, *p* = 0.7; *F*(1,5) = 0.61, *p* = 0.47, respectively. However, when looking at judges’ scores on the combined technical subscales (Pitch/Intonation, Tempo and Rhythm, Vocal Tone and Projection, Musicality) vs. the combined non-technical subscales (Character and Story Telling, Confidence and Stage Presence) for students’ a cappella performances only, the p -value for the effect of time on the students’ non-technical scores [*F*(1,5) = 2.07, p = 0.21] was noticeably smaller than that for the combined technical scales [*F*(1,5) = 0.27, *p* = 0.63]. In particular, the effect of time on judges’ Character and Story Telling scores for students’ a cappella performances was approaching significant [F(1,5) = 3.63, *p* = 0.12] with good inter-rater reliability: ICC (2, 2) = 0.85. However, the effect of time on judges’ scores on the same subscale for students’ accompanied performances was not approaching significant [*F*(1,5) = 1.55, *p* = 0.27]. In general, when looking at the effect of time on judges’ ratings for students’ performances, the *p* -values tended to be smaller for students’ a cappella performances than their accompanied ones, and for the non-technical subscales compared to the technical subscales (Table 3).

**TABLE 3:** Results of ANOVA tests for the main effect of time on three judges’ ABRSM subscale scores for students’ accompanied and a cappella performances, and results of Intraclass Correlation Coefficient analyses using a two-way random model with absolute agreement among the judges.

	Technical subscales			Non-technical subscales	
P/I^1^	T&R^2^	VT&P^3^	M^4^	CH&ST^5^	C&SP^6^
**Accompanied**					
*F*(1,5) = 0.1	*F*(1,5) = 0.25	*F*(1,5) = 0.63	*F*(1,5) = 0.02	*F*(1,5) = 1.55	*F*(1,5) = 0.3
*p* = 0.76	*p* = 0.64	*p* = 0.47	*p* = 0.89	*p* = 0.27	*p* = 0.61
ICC = 0.79	ICC = 0.72	ICC = 0.9	ICC = 0.85	ICC = 0.88	ICC = 0.89
**A Cappella**					
*F*(1,5) = 0.58	*F*(1,5) = 0.00	*F*(1,5) = 0.03	*F*(1,5) = 0.87	*F*(1,5) = 3.63	*F*(1,5) = 0.93
*p* = 0.48	*p* = 1.00	*p* = 0.87	*p* = 0.39	*p* = 0.12	*p* = 0.38
ICC = 0.71	ICC = 0.69	ICC = 0.82	ICC = 0.84	ICC = 0.85	ICC = 0.87

*^1^Pitch/Intonation; ^2^Tempo and Rhythm; ^3^Vocal Tone and Projection; ^4^Musicality (Dynamics, Vocal Expression); ^5^Character and Storytelling; ^6^Confidence and Stage Presence.*

### Changes in Shame Over Having Music Performance Anxiety

Within-group changes on mean scores for a questionnaire measuring state shame (ESS) administered immediately after students’ accompanied and a cappella performances at both pre- and post-coaching were evaluated, using paired *t-*tests. Significant improvements were observed in students’ ESS scores at post-coaching for both types of performances, thereby supporting Hypothesis #3a. ESS scores for students’ post-coaching accompanied performances [*M* = 2.82, *SD* = 0.85] were significantly lower than ESS scores at their pre-coaching accompanied performances [*M* = 5.13, *SD* = 1.1; *t*(5) = −6.39, *p* < 0.05], and similarly, ESS scores for their post-coaching a cappella performances [*M* = 2.47, *SD* = 0.64] were significantly lower than ESS scores at their pre-coaching a cappella performances [*M* = 4.53, *SD* = 1.38; *t*(5) = −4.42, *p* < 0.05]. See Table 2 for effect sizes related to changes in ESS mean scores. Within-group changes on mean scores for a questionnaire measuring the tendency to make unfavorable (upward) comparisons between oneself and others (SCS) were also evaluated, using paired *t*-tests. Significant improvements were observed in students’ SCS scores at post-coaching [*M* = 58, *SD* = 15.14; *t*(5) = 5.37, *p* < 0.05] and at the 3-month follow-up [*M* = 64.5, *SD* = 14.61; *t*(5) = 6.76, *p* < 0.05], when compared to students’ SCS scores at pre-coaching [*M* = 34.17, *SD* = 17.89]. Thus, Hypothesis #3b was also supported. See Table 2 for effect sizes related to changes in SCS scores.

### Changes in Music Performance Anxiety Symptoms

Within-group changes on mean scores for a MPA-based measure (KMPAI) were evaluated at post-coaching and at the 3-month follow-up, using paired *t*-tests. Significant reductions were observed between students’ pre-coaching scores [*M* = 161.83, *SD* = 19.66] and their scores at post-coaching [*M* = 117.67, *SD* = 26.91; *t*(5) = –6.36, *p* < 0.05] and at the follow-up assessment, [*M* = 106.17, *SD* = 24.83; *t*(5) = –6.85, *p* < 0.05] thereby supporting Hypothesis #4. Students’ mean KMPAI score at the 3-month follow-up (106.17) fell very close the author’s recommended cutoff score (105) for determining clinically significant levels of MPA (Ackermann et al., 2014). Furthermore, an independent samples *t*-test showed there were no significant differences between students’ KMPAI scores at the screening assessment [*M* = 160.5, *SD* = 26.81] and at Session 1 [*M* = 161.83, *SD* = 19.66; *t*(10) = –0.1, *p* = 0.46].

### Similarities Between This Study’s Results and Those From Juncos and Colleagues’ ACT for Music Performance Anxiety Psychotherapy Study

**FIGURE 2:**
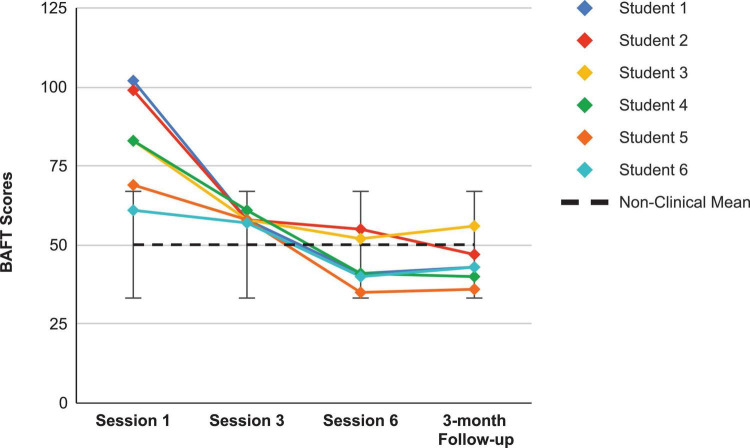
Believability in Anxious Feelings and Thoughts scores for the current students, from pre-coaching (Session 1), midway (Session 3), post-coaching (Session 6), and the 3-month follow-up, with the non-clinical mean (50.10) and bars spanning one standard deviation up/down (*SD* = 16.88; Herzberg et al., 2012).

**FIGURE 3:**
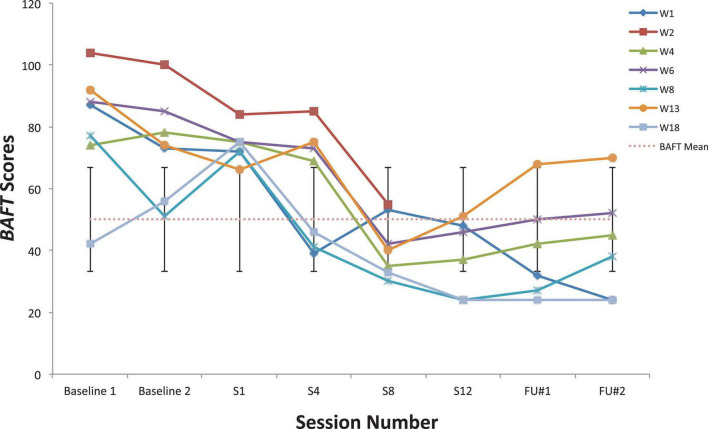
Believability in Anxious Feelings and Thoughts scores for seven vocal students (Juncos et al., 2017) showing scores from the baseline period to post-treatment, 1- and 3-month follow-up points, also with the non-clinical mean (50.10) and bars spanning one standard deviation up/down (*SD* = 16.88; Herzberg et al., 2012).

**FIGURE 4:**
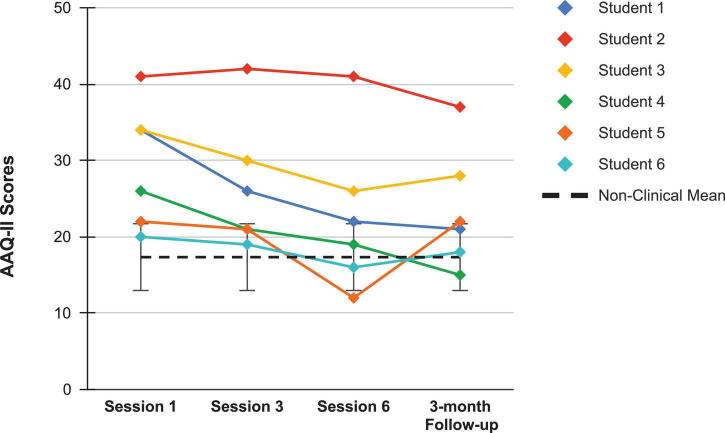
AAQ-II scores for the current students, from pre-coaching (Session 1), midway (Session 3), post-coaching (Session 6), and the 3-month follow-up, with the non-clinical mean (17.34) and bars spanning one standard deviation up/down (*SD* = 4.37; Bond et al., 2011).

**FIGURE 5:**
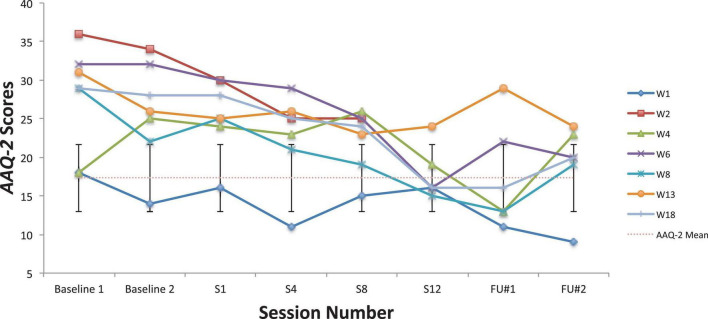
AAQ-II scores for seven vocal students (Juncos et al., 2017) showing scores from the baseline period to post-treatment, 1- and 3-month follow-up points, also with the non-clinical mean (17.34) and bars spanning one standard deviation up/down (*SD* = 4.37; Bond et al., 2011).

**FIGURE 6:**
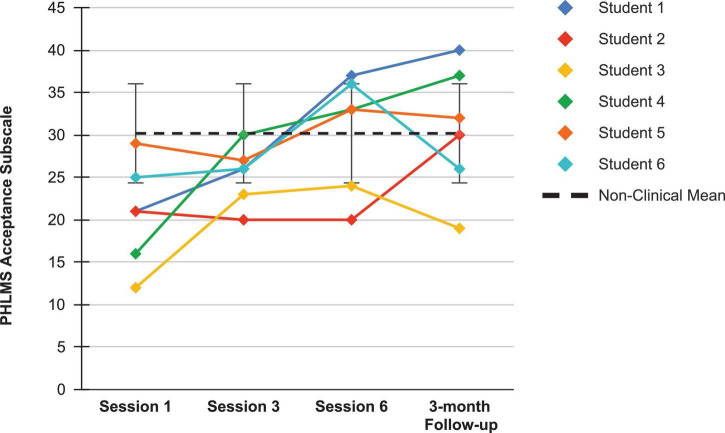
PHLMS Acceptance subscale scores for the current students, from pre-coaching (Session 1), midway (Session 3), post-coaching (Session 6), and the 3-month follow-up, with the non-clinical mean (30.19) and bars spanning one standard deviation up/down (*SD* = 5.84; Cardaciotto et al., 2008).

**FIGURE 7:**
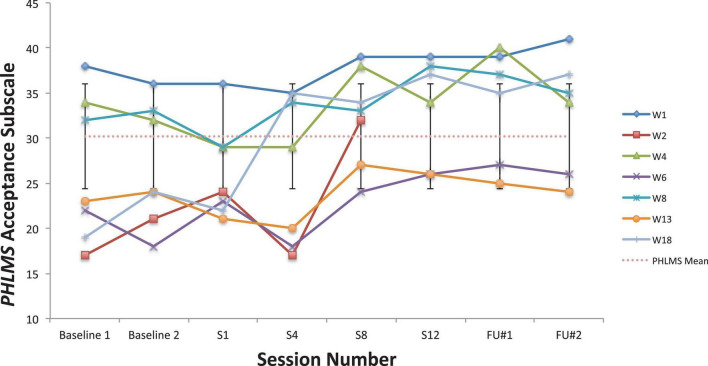
PHLMS *Acceptance* subscale scores for seven vocal students (Juncos et al., 2017) showing scores from the baseline period to post-treatment, 1- and 3-month follow-up points, also with the non-clinical mean (30.19) and bars spanning one standard deviation up/down (*SD* = 5.84; Cardaciotto et al., 2008).

**FIGURE 8:**
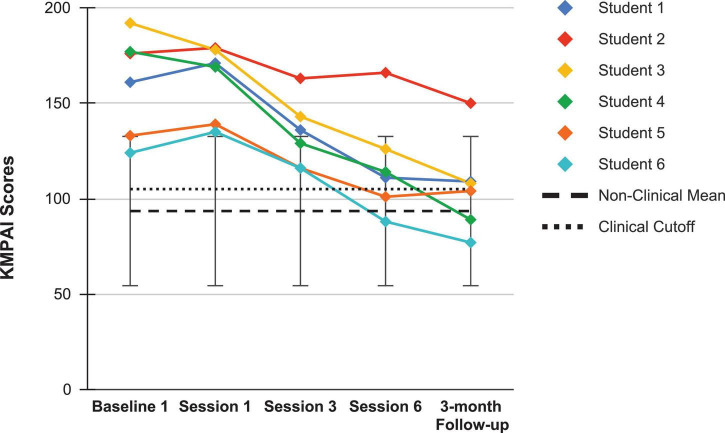
Kenny Music Performance Anxiety Inventory scores for the current students, from the screening assessment (Baseline 1) pre-coaching (Session 1), midway (Session 3), post-coaching (Session 6), and the 3-month follow-up, with the mean for musicians under age 30 (93.5), standard deviation bars (*SD* = 39.1; Kenny et al., 2012), and the recommended clinical cutoff score (105; Ackermann et al., 2014).

**FIGURE 9:**
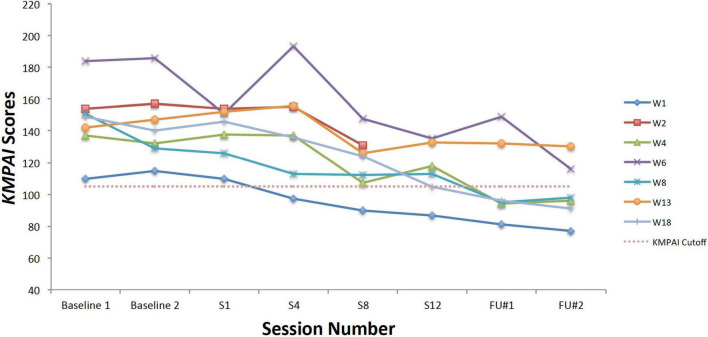
Kenny Music Performance Anxiety Inventory scores for seven vocal students (Juncos et al., 2017) showing scores from the baseline period to post-treatment, 1- and 3-month follow-up points, also with the mean for musicians under age 30 (93.5), standard deviation bars (*SD* = 39.1; Kenny et al., 2012), and the recommended clinical cutoff score (105; Ackermann et al., 2014).

**FIGURE 10:**
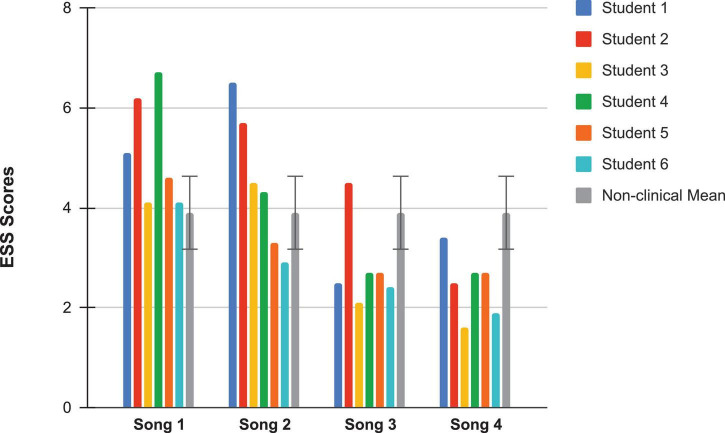
Experiential Shame Scale scores for the current students, from pre-coaching with accompaniment (Song 1), pre-coaching a cappella (Song 2), to post-coaching with accompaniment (Song 3), to post-coaching a cappella (Song 4), with the non-clinical mean (3.9) and bars spanning one standard deviation up/down (*SD* = 0.73; Turner, 2014).

**FIGURE 11:**
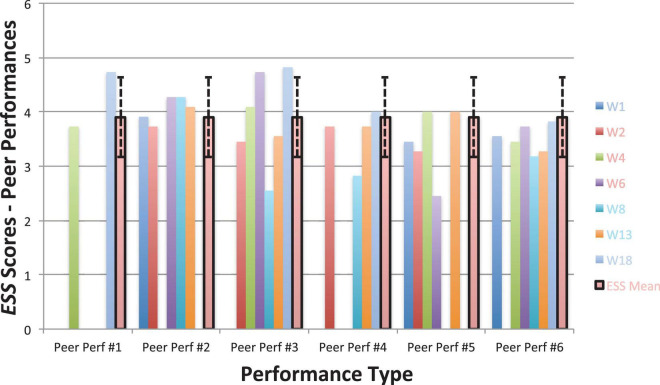
Experiential Shame Scale scores for seven vocal students from six peer performances with piano accompaniment (Juncos et al., 2017), also including the non-clinical mean (3.9) and standard deviation bars (*SD* = 0.73; Turner, 2014).

For a visual inspection of the shared questionnaire results from both studies, the reader is guided from Figures 2 to 11. Similar to Juncos et al. (2017) study, all students in this study (6 of 6) made clinically significant improvements on *at least one* of the shared self-report measures after coaching, whereas all students (7 of 7) made such an improvement in Juncos and colleagues’ study. Here, a *clinically significant* improvement meant a student’s score fell outside the normative range for a questionnaire before coaching and within that range after coaching ended - either immediately afterward (at Session 6) or at the 3-month follow-up point. Such a metric is commonly used when assessing change in single-case designs (Kazdin, 2011). The PHLMS *Awareness* subscale was excluded when conducting this analysis, as students’ mindful awareness was not predicted to improve after the ACC coaching. For the KMPAI, a clinically significant improvement meant either of the students’ baseline scores fell above the recommended cutoff score (105) and below the cutoff at either Session 6 or at the follow-up.

Looking first at the graphs for ACC-based questionnaires common to both studies, Figure 2 shows the current students’ BAFT scores and should be viewed alongside Figure 3, which shows the 7 vocal students’ BAFT scores from Juncos et al. (2017) study. Figure 2 shows the majority of the current students’ scores (5 of 6) fell above the BAFT’s normative range prior to ACC intervention, whereas none of their scores fell within that range at post-coaching (Session 6) or at the follow-up. Similarly, Figure 3 shows all the students’ BAFT scores (7 of 7) in Juncos et al. (2017) study fell above the normative range at baseline or at the beginning of ACT psychotherapy, and all of their scores fell within the normative range (or better) at post-treatment (Session 12). BAFT scores falling above that range indicate higher levels of fusion with MPA-related thoughts and feelings. Figures 4, 5 show the current students’ AAQ-II scores and the vocal students’ AAQ-II scores from Juncos et al. (2017), respectively. Figure 4 shows the majority of the current students’ scores (5 of 6) fell above the AAQ-II’s normative range prior to receiving ACC, whereas the majority (4 of 6) fell within the normative range at Session 6 (or better) and half were within that range at follow-up (3 of 6). Similarly, Figure 5 shows the majority of the vocal students’ AAQ-II scores (6 of 7) fell above the normative range at baseline or at the beginning of ACT psychotherapy, whereas the majority (5 of 6) fell within the normative range at post-treatment and also within that range (or better) at the 3-month follow-up (4 of 6). AAQ-II scores falling above the normative range indicate higher levels of psychological inflexibility. Figures 6, 7 show the current students’ PHLMS *Acceptance* subscale scores and the vocal students’ PHLMS *Acceptance* subscale scores from Juncos et al. (2017), respectively. Figure 6 shows the majority of the current students’ scores (4 of 6) fell below the PHLMS *Acceptance* subscale’s normative range prior to receiving ACC, whereas the majority (5 of 6) fell within the normative range at Session 6, and the majority (5 of 6) also fell within that range (or better) at the follow-up. Similarly, Figure 7 shows the majority of the vocal students’ PHLMS *Acceptance* subscale scores (4 of 7) fell below the normative range at baseline or at the beginning of ACT psychotherapy, whereas all the students’ scores fell within the normative range (or better) at Session 12 and the majority of scores (5 of 6) were within that range (or better) at the 3-month follow-up. PHLMS *Acceptance* scores falling below the normative range indicate lower levels of acceptance of MPA symptoms.

Looking next at the graphs for symptom-based questionnaires common to both studies, Figure 8 shows the current students’ KMPAI scores and should be viewed alongside Figure 9, which shows the seven vocal students’ KMPAI scores from Juncos et al. (2017). Figure 8 shows all of the current students’ KMPAI scores at both baseline points fell above the recommended clinical cutoff score (105), whereas 2 students’ KMPAI scores were below the cutoff at Session 6 and half (3 of 6) were below it at the follow-up. Similarly, Figure 9 shows all of the vocal students’ KMPAI scores were above the cutoff (105) at baseline or at the beginning of ACT psychotherapy (Juncos et al., 2017), whereas 2 students were at or below the cutoff at Session 12 and the majority (4 of 6) were below it at the 3-month follow-up. KMPAI scores falling about the cutoff indicate clinically elevated levels of MPA. Lastly, Figures 10, 11 show the current students’ ESS scores and the 7 vocal students’ ESS scores from when performing in front of their peers in Juncos et al. (2017), respectively. Please note, the ESS scores collected during the vocal students’ peer performances in 2017 (as shown here in Figure 11) are different from the ESS scores taken before/after ACT treatment in Juncos et al. (2017) – the former were included here to compare this study’s clinically significant results to those from Juncos et al. (2017). Figure 10 shows half (3 of 6) the current Students’ ESS scores fell above the normative range prior to the ACC intervention (songs 1 and 2 were recorded pre-coaching, whereas songs 3 and 4 were recorded post-coaching), whereas all ESS scores fell within the normative range (or better) at post-coaching. Similarly, Figure 11 shows two of the vocal Students’ ESS scores from Juncos et al. (2017) fell above the normative range at either their first or second peer performance, yet scores from each student’s final performance fell within the normative range (or better). ESS scores falling above the normative range indicate elevated levels of state shame during music performances.

### Students’ Impressions of the Acceptance and Commitment Coaching Course and Coded Qualitative Data

Throughout the ACC course, SM recorded and transcribed the sessions and then highlighted key statements made by the students detailing their struggles with MPA and their responses to ACC processes and to the overall course. What follows below is a summary of their impressions of the ACC course, as noted by SM during it and at the post-coaching interview. Due to time constraints, the transcripts did not go through the process of member checking, with it being unlikely that the many hours worth of transcribed data would have been returned on time. However, the stringent cross-checking of students’ accounts from multiple sources of qualitative data ensured an accurate representation of how they felt about the process.

They found it useful to have a different topic to focus on each session, “*I liked having something to work on each week. And then…it gradually built up.*” The small group size was related to an increased sense of social inclusion and support, “*Everyone got comfortable with each other because it was a small group…you don’t feel intimidated.”* This sense of belonging was related to reduced shame over having MPA, “*It was useful to relate to others…hear them saying things I felt…realizing you’re not alone. Sometimes I used to think I was the only one.*” The first session included information and discussion about why some performers experience MPA. Students reported the “*learning about the biological background of these symptoms made it easier for me [them] to accept them and not judge myself [themselves] for any feelings*,” and the group dialogue helped normalize MPA, leading students to realize that “*MPA is not talked about enough.*”

Prior to ACC, students engaged in overt and covert avoidant behaviors to manage their MPA, “*I often give up on songs and discard them as the negative thoughts dominate*,” “*I used to, like, avoid singing as much as possible and like, be ill, so that I wouldn’t have to sing.*” They found themselves fused with perfectionistic and self-critical thoughts, “*If I made one tiny mistake, I would beat myself up*,” and they were frustrated with MPA symptoms serving as barriers to progress with their singing, “*I work a lot on my songs but feel people can never tell. It makes me really frustrated, because I know that I can do it. But then when I get to do it in front of someone, I can’t.*”

They were able to implement tools and techniques from ACC into their lessons and performances almost immediately, “*I used mindful breathing before singing practice and my singing lesson and I was less tense.*” One student remarked that present moment awareness had a positive effect on their vocal technique, “*When I got stressed about singing the high notes…I was able to focus on something (in the room) to bring me back to the present, and I found that I could sing them!*” They reported the defusion techniques were particularly helpful too, giving them instant, practical tools, e.g., saying their thoughts aloud in a silly voice or character. They found these exercises imaginative and fun, which resonated with their values as performers, “*I got anxious before singing in front of the class…I put on a silly voice and sung my thoughts, which made me laugh. Then I felt more relaxed, more grounded.*” Defining their performance-related values gave students a sense of meaning and direction, enabling them to accept MPA symptoms while continuing to perform, “*This week was the first time I had sung in front of the class…it had been building up. But I decided I would take valued action despite the nerves – it went better than I thought!*”

The work on self-compassion appeared to be a major turning point in the students’ progress. For most, this was an alien concept, as they felt they could only be highly critical of themselves. They defined barriers to self-compassion, such as not being deserving, “*I tell myself I can’t be kind to myself as I don’t deserve it*,” fear of being perceived as arrogant, and the need to be perfect. However, the work on self-compassion led to realizations such as, “*You would never speak that way to a friend. Maybe I could talk to myself before a solo like I would talk to my best friend*,” and “*If you had self-compassion after a performance, you’d be accepting of it…you might even be proud*.” This work appeared to improve their overall wellbeing as well, “*I bought a gratitude journal as part of self-compassion element…I want to be more positive and make lifestyle changes.*”

They were motivated by supportive feedback from their individual singing teachers, who had noticed improvements in their singing, “*My singing lesson actually was really good…and I got compliments saying he [the teacher] could see real progress*,” and “*Even after the second week, within my singing lessons my teacher would say ‘*Your mindset’s completely different already.’” Following the coaching, students were more accepting of their MPA symptoms, and more willing to perform in their presence, “*I don’t need to force them [the symptoms] to stop, I can still perform.*” They also demonstrated improved psychological flexibility by using their ACC skills in combination with each other, “*I noticed comparison thoughts in class, remembered my values, and moved toward them. Less went wrong, and when it did, I moved on.*” They made connections between ACC for singing and other disciplines, such as dance, “*Before my ballet assessment I used [ACC exercises] and it works just the same as doing it before singing*.” Their written feedback at the follow-up interview suggested they experienced lasting change, and that the overall ACC course was very helpful for them. Some remarks were, “*It’s changed my approach to singing lessons*,” “*Being more accepting of my journey lifted a lot of pressure off my shoulders*,” and “*It’s not just helped me from a singing perspective, I feel like taking the things I’ve learned from this about singing and performance, and then add it to life.*”

**FIGURE 12:**
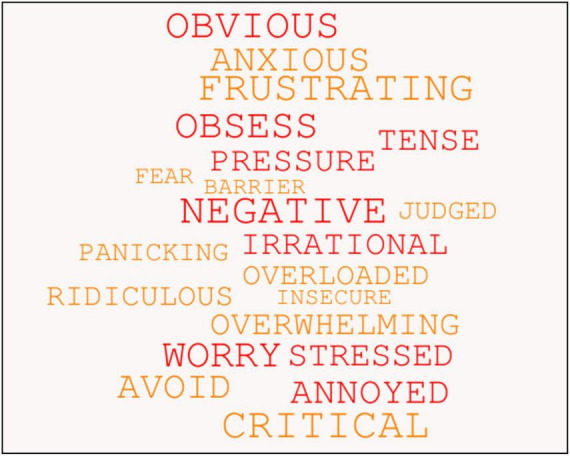
Students’ use of psychologically inflexible language at Session 1, as revealed through emotion coding of their verbatim qualitative data.

**FIGURE 13:**
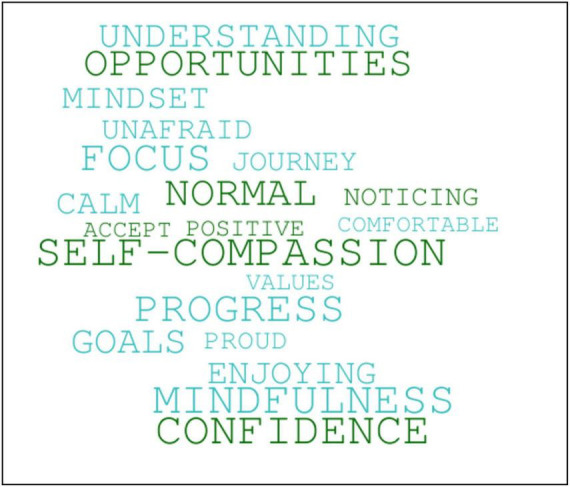
Students’ use of more psychologically flexible language at Session 6, as revealed through emotion coding of their verbatim qualitative data.

In ACT/ACC, the use of language and cognition is contextual, and as the mindset of the students changed during the coaching intervention so too did their use of language. This change correlated with them moving away from psychologically inflexible behavior and toward more psychologically flexible behavior. Using emotion coding to look at students’ verbatim qualitative data at Session 1 (Figure 12) and Session 6 (Figure 13), a visual snapshot emerged of their changing emotional states from pre- to post-coaching.

## Discussion

Consistent with Shaw et al. (2020) findings, the results obtained here provide further preliminary support for an alternative, non-clinical treatment model for MPA – that is, it appears possible a singing teacher without training or education in psychotherapy may achieve significant results, and seemingly replicate the results obtained by a clinical psychologist (Juncos et al., 2017), when trained to use ACC to help manage MPA for a small group of performing arts students with anxiety related to their vocal performances. Such an intervention could be feasible in fulfilling the growing need for music teacher training in support of students who suffer with problematic MPA, as identified by a number of researchers (Fehm and Schmidt, 2006; Patston, 2014; Patston and Waters, 2015; Sarikaya and Kurtaslan, 2018; Jeong and Ryan, 2021). If the results observed here were valid, the six students showed a very similar pattern of improvement as both the musical theatre student did in Shaw et al. (2020) study, and the seven vocal students did in Juncos et al. (2017) study. After six group ACC sessions, they appeared to make significant improvements in their ability to mindfully accept physiological MPA symptoms, defuse from MPA-related cognitive symptoms, and perform in a more psychologically flexible manner, even while experiencing distressing MPA symptoms. The observed improvements in these ACC processes were also maintained at the 3-month follow-up assessment. The processes of acceptance of distressing experiences and defusion from them are two proposed mechanisms by which ACT treatments enhance psychological flexibility (Ciarrochi et al., 2010), and these changes have been observed with other musicians who received ACT psychotherapy for MPA (Juncos et al., 2014; Juncos and Markman, 2015; Clarke et al., 2020). The singing teacher in this study received training in ACC from the second author that was of a similar duration and format (8 h via Zoom) as the training administered in Shaw et al. (2020) study (7 h via Skype), which suggests a brief training in ACC of less than 10 h may be sufficient to produce significant results for singing teachers, and perhaps music teachers at large, who are looking to address students’ problematic levels of MPA.

In studies with stronger internal validity than the current one, other applications of ACT have led to significant improvements in psychological flexibility when delivered by non-expert practitioners of varying professions, which lends further credibility for a non-clinical treatment model for MPA. For example, 40 early childhood teachers with low singing confidence were randomized to receive either four group sessions of ACT or four sessions of group singing (Swain and Bodkin-Allen, 2017). A trained singer was selected to facilitate all the sessions, because her knowledge of vocal technique was relevant for the group singing intervention, and she was able to be trained to deliver the ACT sessions effectively. Both the ACT and group singing interventions significantly improved the teachers’ singing confidence, as measured by their answers to six questions about overall singing confidence (Swain and Bodkin-Allen, 2017). Another important example from an unrelated profession is how American correctional officers with a Bachelor’s degree, and little to no clinical background, were trained to deliver an ACT-based, group intervention for men with domestic violence offenses (Zarling et al., 2017, 2020). The results showed men in the ACT group (*N* = 843) committed significantly fewer domestic assault charges, or violent charges, than participants receiving a group Cognitive Behavioral Therapy intervention (*N* = 2,361) at both a 1-year and 5-year follow-up point. The officers’ training included 4 days of ACT training plus ongoing supervision throughout the intervention with non-clinical peers already trained in ACT (Zarling et al., 2017, 2020). Furthermore, a recent, well-controlled study investigated ACT’s effectiveness as an adjunct to physical therapy for treating chronic lower back pain (Godfrey et al., 2020). Two hundred and forty eight adults with chronic lower back pain were randomly assigned to receive either ACT-informed physical therapy or physical therapy alone, and the results showed the ACT-informed group reported significantly better outcomes than the physical therapy group for pain-related disability, physical health, and patient functioning at a 3-month follow-up (Godfrey et al., 2020). The ACT interventions were administered by physical therapists who were trained to achieve a high fidelity to the ACT model. In fact, when it comes to chronic pain treatment, England’s National Health Service (NHS) now recommends using psychologically informed practices to treat chronic pain, when other treatments are proven ineffective or when a condition is at risk for becoming chronic (de Campos, 2017; National Health Service of England, 2017). Considering the preliminary results observed in the current study, those from Shaw et al. (2020), and the results of these other non-psychotherapist delivered applications of ACT, there is a growing consensus that adequately trained practitioners other than psychotherapists may be capable of effectively delivering psychological interventions, despite their feeling unqualified to do so (Hall et al., 2018). However, future research with larger samples of teachers and stronger internal validity is needed first to determine whether the observed results across some of the studies are indeed due to the ACT/ACC interventions. If teachers can demonstrate efficacy in applying these interventions, recommendations for psychologically informed practices should be incorporated into curriculum guidelines for performing arts and music colleges as well, because MPA, like chronic pain, is a potentially chronic and debilitating condition if untreated, and psychotherapy may be an unrealistic treatment for it considering the aforementioned hurdles preventing music students from seeking psychotherapeutic help. Training music teachers to deliver ACC-based interventions individually and/or in group settings appears ecologically valid and is possibly more acceptable for students than referring them to a psychotherapist.

The students here reported experiencing significantly less states of shame during their performances at post-coaching, and significantly less MPA at post-coaching and at a 3-month follow-up, as the seven vocal students also did in Juncos et al. (2017) study. Additionally, a novel finding here was the students significantly improved their socially comparative thinking at post-coaching and follow-up. These findings are potentially noteworthy, considering the students in Juncos et al. (2017) study received 12 ACT psychotherapy sessions whereas the students here received six group ACC sessions. It appears possible, then, to achieve significant reductions in MPA and in one’s emotional distress related to vocal performance in less time than previously thought. One possible explanation for a shorter ACC intervention leading to significant reductions in MPA and less maladaptive use of social comparisons was through improvements in defusion skills occurring *prior* to the reductions in those symptoms. As shown in Figure 4, all students’ scores on the BAFT clearly fell within a healthy range by Session 3, which was the point in the ACC course when SM taught them defusion techniques. By that time, students appeared to learn how to notice the occurrences of their unwanted, cognitive MPA symptoms and their unhelpful comparisons to others, without taking them as literally or personally, and possibly enabling themselves to perform more flexibly in their presence. Table 2 also shows a very large effect size with respect to students’ improvements on BAFT scores was found at post-coaching (*g* = 3.07), yet it remained the same at the follow-up point (*g* = 3.07). On the other hand, the effect sizes for students’ improved scores on the KMPAI and SCS were both notably larger at follow-up than at post-coaching: *g* = 1.87 at post-coaching and 2.48 at the follow-up for changes in KMPAI scores; *g* = 1.44 at post-coaching and 1.86 at the follow-up for changes in SCS scores. These different trends suggest the ACC intervention was exerting a greater effect on students’ MPA symptoms and maladaptive social comparisons *after* the coaching had ended, compared to its effect on their defusion skills during that same timeframe. Such an outcome was also observed in Juncos et al. (2017) study with the vocal students’ KMPAI scores: *g* = 1.55 at post-treatment and *g* = 2.19 at a 3-month follow-up. Thus, it is possible students in both studies were continuing to defuse from their unwanted symptoms post-intervention, which led them to suffer less with their symptoms by a 3-month follow-up point. Of course, there could be other reasons for the continued reductions in MPA symptoms and improved social comparisons after the coaching finished, i.e., regression to the mean, students feeling more relaxed as their fall semester had recently ended. However, such temporal differences in outcomes have been observed in numerous ACT treatments, and consequently, there is a growing body of support for the hypothesis that changes in psychological flexibility, via improvements in defusion (as well via improvements in acceptance and increased valued actions) will precede reductions of symptoms in both clinical samples (Dalrymple and Herbert, 2007; Twohig et al., 2010; Kocovski et al., 2013; Juncos and Markman, 2015; Gloster et al., 2017; Juncos et al., 2017) and non-clinical samples (Zettle and Hayes, 1986; Gifford et al., 2004; Hesser et al., 2009). Therefore, the length of the ACC intervention may not predict reductions in symptoms as much as the prerequisite that improvements in psychological flexibility occur first, regardless of the duration of the intervention.

In reference to the second hypothesis, there was not a significant improvement in the quality of students’ accompanied or a cappella vocal performances at post-coaching, according to the three judges’ ratings. This could be due to the small sample of students and small number of judges, and also to the judges’ inexperience in using the ABRSM scoring criteria, though to a lesser extent. A future ACC for MPA study should investigate this same hypothesis with a larger sample of students, as many as four or five judges, and with judges more experienced in adjudicating vocal performances with the ABRSM criteria. It is possible the observed improvements in scores for one non-technical subscale, i.e., Character and Story Telling, may become significant with a larger sample of students and/or more judges. Such a potential outcome would make sense, considering ACC does not aim to improve the technical elements of vocal performance, but it would be expected to help students lessen their struggle with MPA symptoms and maladaptive social comparisons, which could potentially free up more energy to be put into their valued-actions like communicating their connection to the character and text through their performances. In fact, ACT treatments have led patients with various anxiety conditions to earn better ratings from independent observers regarding the quality of their performances on various behavioral tasks evoking fear, e.g., a public speaking task for patients with public speaking anxiety (Glassman et al., 2016), an academic test for students with test anxiety (Brown et al., 2011), a distress tolerance task for patients with spider phobia (Wagener and Zettle, 2011), a social interaction task for patients with Social Anxiety Disorder (Herbert et al., 2018), a music performance for an undergraduate violinist with MPA (Juncos and Markman, 2015), and accompanied/a cappella performances for seven vocal students with MPA (Juncos et al., 2017). Furthermore, ACT-related interventions have also led to improved self and observer ratings of performances when applied non-clinically in athletic, academic, and occupational settings (Bond and Bunce, 2003; Lutkenhouse et al., 2007; Bernier et al., 2009; Thompson et al., 2011; Ruiz and Luciano, 2012; Castro et al., 2016; Paliliunas et al., 2018; Josefsson et al., 2019). All of these aforementioned studies included objective measurements of participants’ performances, and most included samples with more than six participants, with the exceptions of Ruiz and Luciano (2012), Juncos and Markman (2015), and Castro et al. (2016). As mentioned earlier, the judges’ inexperience with using the ABRSM criteria could have led to the observed non-significant improvements in students’ performance quality as well. However, the judges’ inter-rater agreement ranged from moderate to excellent (Table 3), and they had extensive experience assessing vocal performances using similar criteria in musical theatre colleges, thus, their use of the ABRSM scoring tool was deemed to be reliable. Lastly, it was unclear why the students’ a cappella performances might have earned them better ratings on the Character and Story Telling subscale at post-coaching than their accompanied performances did on the same subscale. One possibility is that their accompaniment was not typical for them – given the UK’s restrictions on social gatherings in late 2020 due to the COVID-19 pandemic, students were neither able to perform in-person nor with a live pianist, so they provided an instrumental back-up track instead. It is likely they lacked experience with this type of accompaniment, and the judges had less experience adjudicating such performances, and perhaps these factors led to a difference in how a cappella vs. accompanied performances were rated.

One final consideration when interpreting the results is that the group delivery of ACC could be responsible for some of the improvements observed. While Juncos et al. (2017) also included group performances in the second half of their ACT psychotherapy study – those students performed a minimum of four times in front of their peers who were also participants in the study, with some students performing six times – there were no group discussions of MPA and ACT-based processes in that study. Rather, those discussions occurred privately in students’ psychotherapy sessions (Juncos et al., 2017). Communication about MPA may prove more beneficial in a group setting, because several known processes that facilitate individual psychological and behavioral change occur more readily in groups, i.e., social comparisons, social support, the provision of challenging yet supportive feedback (Borek and Abraham, 2018). It is possible, then, that learning about MPA in a group setting, and hearing others’ experiences with it, provided students here with the social support needed to create a sense of common suffering with MPA that mitigates against the distressing effects of MPA and unhelpful social comparisons. Providing a sense of common humanity like this helps to buffer against the harmful effects of self-criticism and isolating oneself when attempting to manage mental health problems like anxiety and depression (Gilbert and Procter, 2006; Neff, 2012; Neff and Germer, 2013). It is reasonable that such emotional support would be fostered more easily in a group setting than an individual one. In support of this, the effect sizes obtained here for students’ changes on the KMPAI at post-coaching and the 3-month follow-up were larger compared to those obtained in the 2017 study: post-coaching (*g* = 1.87) and follow-up here (*g* = 2.48), compared to post-treatment (*g* = 1.55) and follow-up (*g* = 2.19) in Juncos et al. (2017) study. The same was true for the effect sizes for students’ changing ESS scores on their accompanied vocal performances: post-coaching here (*g* = 2.35), compared to post-treatment (*g* = 2.03) in Juncos et al. (2017) study. The combination of group discussions about MPA and group performances may have contributed to the slightly larger effect sizes obtained here on students’ ESS scores compared to those from Juncos et al. (2017) study. Moreover, it is also possible the group delivery of ACC led students to make significant improvements in their ability to accept physiological MPA symptoms, a finding that was not observed as robustly with the students in Juncos et al. (2017) study. See Table 1 here for changes in students’ PHLMS *Acceptance* data, and see Table 1 in Juncos et al. (2017) study for similar data. Thus, providing supportive, yet challenging, feedback about the futility of attempting to control one’s MPA symptoms may prove more beneficial when communicated in a group setting than when communicated individually.

### Limitations and Future Directions

Given this study had a small sample and lacked a control/comparison group, direct conclusions about ACC’s efficacy as an intervention for problematic MPA cannot be made. Without stronger checks on internal validity, potential confounds such as history, regression to the mean, and maturation could not be ruled out as alternative explanations of the results. It is possible, for example, the students became less anxious toward the end of the ACC course because their winter break was starting at that time, and also that their scores on the self-report questionnaires were regressing to each measure’s mean by post-coaching. Both of these confounds could explain why their scores had improved after the ACC intervention. However, the apparent similarities between the results observed here and those from previous ACC/ACT for MPA studies (and from other ACT for anxiety studies) strengthen the likelihood the observed improvements in self-report data were due to the ACC intervention. Furthermore, it is possible the non-significant improvements on the Character and Story Telling subscale on the ABRSM criteria were due to students’ ongoing engagement with their broader educational curriculum, rather than to the ACC intervention. Students are expected to make improvements in their performance skills over time, which theoretically would include both technical and non-technical improvements. However, considering that none of the judges’ ratings on the four technical subscales from the ABRSM criteria were approaching significant at post-coaching (Table 3), and that ACC/ACT would be expected to improve the more non-technical, or behavioral, elements of music performance with its emphasis on increasing valued actions in spite of MPA, it is possible the non-significant improvement on the Character and Story Telling subscale may be due to the ACC course. Future studies with larger samples and more judges are needed to establish whether ACC/ACT treats MPA via some of the mediating processes observed here, i.e., defusion, acceptance, and whether it differentially affects the technical vs. non-technical elements of students’ performances.

One potential difficulty in replicating this work in music and performing arts colleges will be in overcoming some practical challenges in applying it. It may be difficult for singing teachers (and students) to carve out an additional 6 h a semester to administer a group ACC course to students with MPA, on top of their already-busy schedules. Some students also may feel uncomfortable with the group nature of the work, yet they may still struggle with problematic MPA and shame so they may avoid participating, unfortunately. It remains to be seen whether other, more realistic ACC interventions may also help music teachers effectively manage students’ MPA, e.g., delivering a didactic-only ACC intervention in a classroom setting to potentially impact more students but without a coaching element, or incorporating ACC interventions more “on the fly” within private lessons, rather than dedicating an hour each week for 6 weeks, as was done here and in Shaw et al. (2020) study. Future studies should examine the effectiveness of such interventions that may be easier to deliver for a larger number of music teachers. However, given the consistency between the results obtained here and those obtained by a clinical psychologist in Juncos et al. (2017), it is possible a group ACC course would continue to benefit students struggling with MPA and shame-related distress. For those schools who can afford to implement it, the same group ACC course should be replicated to see if it makes a similar impact.

At times, SM needed to consult with the second author, a clinical psychologist with expertise in treating MPA with ACT, about how best to proceed when certain students responded more slowly to the ACC intervention or in other ways that were difficult to understand. A music teacher administering ACC to a similar cohort will inevitably face similar challenges in working with students of varying levels of psychological inflexibility and flexibility. However, not every teacher has access to a psychologist with adequate experience in ACC, or in treating MPA, for consultation about such students, so this aspect of the study may not translate easily into real-world settings. In these instances, we strongly recommend the teacher seek supervision or peer consultation from an ACT-trained practitioner(s), many of whom can be found through the Association for Contextual Behavioral Science’s webpage www.contextualscience.org, which is an online learning and research community for those seeking information about ACT. It is actually common for participants undergoing ACT treatments for anxiety to resist some of its central ideas, i.e., acceptance of anxiety rather than attempting to control it, especially given that avoidant behaviors are negatively reinforced in the short term (Gloster et al., 2017). Unfortunately, there is a predictable pattern between the degree to which one struggles against their anxiety and their emotional suffering related to it, in that patients whose symptoms of anxiety remain high during treatment are likely still engaged in a struggle to control them (Gloster et al., 2017). This may have happened with some students here (see Figures 6, 8, 10).

During their post-coaching interviews, students had asked that more time be dedicated to learning about and improving self-compassion in future group ACC courses, as they appeared to benefit greatly from this work. SM was able to incorporate self-compassion into just one session, as it wasn’t part of the ACC curriculum adopted from Hill and Oliver’s (2019) six-session guide. ACT is highly compatible with other therapies aiming to enhance self-compassion, e.g., Compassion Focused Therapy (Gilbert, 2010) and Mindful Self-Compassion (Neff and Germer, 2013), thus, we recommend future researchers include more opportunities to train in self-compassion within group ACC interventions for music and performing arts students with problematic MPA.

Lastly, the ACC course was conducted entirely online via Zoom due to the UK’s restrictions on social gatherings during the COVID-19 pandemic. Future group ACC courses should be conducted in person to compare the results to those obtained here. It is possible the online environment, and the lack of live accompaniment, created less anxiety for students when performing in front of one another as one might expect from live performances, even though their KMPAI and ESS scores did not appear different than those of the vocal students in Juncos et al. (2017) study. Neither of those two questionnaires measured state anxiety during performances, so future ACC for MPA research should include measures like *The State-Trait Anxiety Inventory* (STAI; Spielberger, 1983) to ensure there are no significant differences between students’ MPA levels when performing live vs. online.

## Conclusion

This mixed-methods pilot study marked the first application of a group ACC course administered by a singing teacher to help manage MPA for a small sample of performing arts students (*N* = 6) with anxiety about their vocal performances. After receiving a brief ACC training of less than 10 h she appeared to replicate the results of a previous ACT for MPA treatment administered by a clinical psychologist (Juncos et al., 2017), and the results of an ACC for MPA single-subject design also administered by a singing teacher (Shaw et al., 2020). However, given the small sample size and lack of a control group, it was unclear if the observed results were due to the ACC intervention. If valid, these results and those from Shaw et al. (2020) study add to a small, but growing body of research supporting the potential effectiveness and feasibility of non-clinical interventions for MPA (Sisterhen, 2005; Hoberg, 2008) and of non-clinical applications of ACT in a broader context (Swain and Bodkin-Allen, 2017; Zarling et al., 2017, 2020; Godfrey et al., 2020; Shaw et al., 2020). Such an alternative model for treating MPA is promising and should be studied further in other forms, as it could be more helpful than psychotherapy for students in music and performing arts colleges with problematic MPA, given the common hurdles preventing them from working with a psychotherapist, i.e., stigma, lack of time/access to services (Shaw et al., 2020). In particular, the combination of group performances and group discussions about MPA appeared to lower students’ levels of shame while performing in front of another slightly better than the combination of individual psychotherapy and group performances did in a previous ACT for MPA study (Juncos et al., 2017), thus, future group ACC interventions should include supportive discussions to help normalize students’ experiences with MPA. The same appeared true for ACC-based group discussions that challenged students’ attempts to control MPA symptoms - students here reported a better ability to accept MPA symptoms than students receiving individual ACT psychotherapy did (Juncos et al., 2017). For an outline of the ACC course used here, please visit: https://www.researchgate.net/publication/358271152_Acceptance_and_Commitment_Coaching_for_MPA_Course_Design_Sarah_Mahony.pdf, and for more information about ACC/ACT, please visit www.contextualscience.org.

## Data Availability Statement

The original contributions presented in the study are included in the article/supplementary material, further inquiries can be directed to the corresponding author/s.

## Ethics Statement

The studies involving human participants were reviewed and approved by University of Wales Trinity Saint David. The patients/participants provided their written informed consent to participate in this study. Written informed consent was obtained from the individual(s) for the publication of any potentially identifiable images or data included in this manuscript.

## Author Contributions

SM and DJ designed the study. SM and DW obtained IRB approval. SM created and administered the group ACC course for the six students. DJ provided the ACC training and ongoing supervision for SM about the sample’s response to the ACC course, and about SM’s quantitative data and issues related to statistical significance. DJ and SM co-wrote and edited the manuscript. DW provided supervision to SM on the study’s ethical matters and on understanding the qualitative data. All authors contributed to the article and approved the submitted version.

## Conflict of Interest

The authors declare that the research was conducted in the absence of any commercial or financial relationships that could be construed as a potential conflict of interest.

## Publisher’s Note

All claims expressed in this article are solely those of the authors and do not necessarily represent those of their affiliated organizations, or those of the publisher, the editors and the reviewers. Any product that may be evaluated in this article, or claim that may be made by its manufacturer, is not guaranteed or endorsed by the publisher.
